# Amplifying the Voices of Latino Immigrant Dementia Caregivers: Using Photovoice to Assess Resources

**DOI:** 10.1093/geroni/igaf122.262

**Published:** 2025-12-31

**Authors:** Mayra Sainz

**Affiliations:** Emory University, Atlanta, Georgia, United States

## Abstract

The high prevalence of dementia among Latino individuals is particularly concerning for Latino immigrants, who on average tend to be older, have longer life expectancies, and are less likely to have health insurance compared to their U.S.-born counterparts. However, the role of immigration in dementia caregiving remains largely underexplored in the literature. This study employed photovoice methods to assess the availability and accessibility of resources for Latino immigrant caregivers of individuals living with dementia. This photovoice study design was guided by the Socio-Ecological Model and consisted of four components: pre-screening interviews, an orientation, photography exercises, and a community forum. The photograph exercises included photograph led discussion employing the SHOWED/VENCER guide. Photographs and transcripts were used to develop a codebook and identifying key themes. Nine immigrant Latino dementia caregivers participated in the photovoice exercise, all of whom were women, with an average age of 55.9 years (range: 38 to 75 years) and reported having lived in the United States for an average of 18.2 years (range: 1 to 32 years). Emerging themes include (1) insufficient support between family members, (2) limited access to mental health services, (3) lack of dementia-friendly environments, (4) the importance of prioritizing sustainability, and (5) a need to increase collaboration opportunities for Latino immigrant caregivers. Research should commit to developing scalable, long-term solutions that integrate cultural factors, enhance mental health support, and expand outreach strategies. Increasing the availability and accessibility of resources for immigrant caregivers will be crucial in fostering a comprehensive caregiving environment for Latino families.

